# Multicentre Study of Chronic Wounds Point Prevalence in Primary Health Care in the Southern Metropolitan Area of Barcelona

**DOI:** 10.3390/jcm10040797

**Published:** 2021-02-16

**Authors:** Miguel Ángel Díaz-Herrera, José Ramón Martínez-Riera, José Verdú-Soriano, Raúl Miguel Capillas-Pérez, Carme Pont-García, Silvia Tenllado-Pérez, Oriol Cunillera-Puértolas, Miriam Berenguer-Pérez, Vicente Gea-Caballero

**Affiliations:** 1 Direcció d’Atenció Primaria Costa Ponent, Institut Català de la Salut, Avinguda de la Gran via de l’Hospitalet, 199-203, 08908 L’Hospitalet de Llobregat, Barcelona, Spain; madiaz@ambitcp.catsalut.net (M.Á.D.-H.); raulmcapillas@gmail.com (R.M.C.-P.); stenlladop@gmail.com (S.T.-P.); 2 Teaching Committee, Hospital Universitari General de Catalunya, 08195 Sant Cugat del Vallès, Barcelona, Spain; 3 Unitat de Suport a la Recerca Costa de Ponent, Institut Universitari d’Investigació en Atenció Primària Jordi Gol (IDIAP Jordi Gol), 08940 Cornellà de Llobregat, Spain; ocunillera.idiap@gmail.com; 4 Community Nursing, Preventive Medicine, Public Health and History of Science Department, Faculty of Health Science, University of Alicante, 03690 Alicante, Spain; miriam.berenguer@ua.es; 5 Universitat Autònoma de Barcelona, 08193 Bellaterra (Cerdanyola del Vallès), Spain; 6 Nursing School La Fe, Adscript Center of University of Valencia, Research Group GREIACC, Health Research Institute La Fe, Avinguda Fernando Abril Martorell nº 106, Torre H. CP, 46006 Valencia, Spain; gea_vic@gva.es

**Keywords:** cross-sectional studies, diabetic foot, foot ulcer, leg ulcer, pressure ulcer, prevalence, primary health care, varicose ulcer

## Abstract

Background: Chronic wounds give rise to major costs and resource consumption in health care systems, due to their protracted healing time. Incidence and prevalence data are scarce or nonexistent in community settings. Objective: The aim of the present epidemiological study was to analyse and determine the prevalence of chronic wounds in the community in the south of the province of Barcelona (Spain). Design: A cross-sectional, multicentre secondary data analysis study was conducted in the community (excluding nursing homes) in Barcelona between 16 April and 13 June 2013. It included 52 primary care centres that serve a total population of 1,217,564 inhabitants. Results: The observed prevalence was 0.11%. Venous ulcers presented the highest prevalence, at 0.04%, followed by pressure injuries, at 0.03%. The >74 age group presented the highest frequency of chronic wounds, accounting for 69.4% of cases. Conclusion: The results obtained are consistent with those reported in previous similar studies conducted in Spain and elsewhere. As with most studies that adjusted their variables for age and sex, we found that the prevalence of ulcers increased with age and was higher in women, except in the case of diabetic foot ulcers and ischaemic ulcers, which were more frequent in men.

## 1. Introduction

A chronic wound is a skin injury that fails to proceed through the normal skin repair response [1]. Due to their protracted healing time, chronic wounds give rise to major costs and resource consumption in health care systems. Pressure injuries (PI) and lower-extremity ulcers (LU) of venous (VLU), ischaemic (IU) or neuropathic aetiology—in the latter case, specifically diabetic foot ulcers (DFU)—overburden community nurses who provide the associated care for patients and family members in clinics and at home [2,3].

Chronic wounds have a great impact on quality of life [4]. Various national health systems now include the implementation of good PI management practices among their patient-safety strategies [5]. However, there are no standardised epidemiological indicators for chronic wound management. Incidence and prevalence data are scarce or nonexistent in community settings, in contrast to hospitals and nursing homes [6,7]. This lack of epidemiological information is compounded by the disparate methods used and results obtained in studies conducted at the community level [8].

Studies on PI prevalence have reported diverse results ranging from 0.031% to 0.11% in the general population and from 6.7% to 12.6% in the population receiving home care [9,10,11,12,13,14]. Studies of LU have attempted to measure prevalence; however, as with other chronic wounds, the varying methods used, and results obtained, render it difficult to establish a value that can be extrapolated beyond the study population. These studies have included different populations (community, nursing home and hospital), various data collection methods and assorted aetiologies. In studies included in a 2019 meta-analysis, LU prevalence ranged between 0.04% and 0.70% [8].

Another meta-analysis conducted in 2003 observed a prevalence of VLU between 0.12% and 0.32% and concluded that due to differences in the study populations, it was inappropriate to group the prevalence rates reported in the various studies [15]. Subsequently, other prevalence studies have been published that share characteristics with lower-extremity ulcer studies, reporting results that range from 0.01% to 0.09% [7,12,16,17,18,19,20].

Chronic wound studies have included the prevalence of DFU in the community. DFU account for approximately 13% of all chronic wounds, a lower percentage than PI or LU [21]. In 2017, Zhang et al. [22] analysed the global prevalence in all types of population and found that men and patients with diabetes mellitus type 2 presented a higher number of cases. They also observed substantial differences between continents (ranging from 13% in North America to 3% in Oceania) and estimated a community prevalence of 2.9% in people with diabetes.

Other studies that have analysed chronic wounds in the community setting with different methodologies show higher prevalence data, ranging between 3.7% and 11.8% [23,24,25].

These widely varying results in the literature hinder extrapolation of the data to other epidemiological assessment systems and thus would hamper the assessment of community care models for people with chronic wounds. Consequently, it is necessary to determine the prevalence of chronic wounds at the local level in different health care systems. This would provide a rationale for conducting further large-scale epidemiological studies in the community.

The aim of the present study was to determine the prevalence of chronic wounds in the community in the south of the province of Barcelona, exploring the demographic and clinical profile of patients with these wounds.

## 2. Materials and Methods

### 2.1. Design

This was a cross-sectional, multicentre, secondary data analysis study.

### 2.2. Study Area and Population

The study was conducted in the community (excluding nursing homes) in the south of the province of Barcelona (Costa de Ponent Primary Care Area), Spain. It included 52 primary care centres managed by the Institut Català de la Salut (Catalan Health Institute) that serve a population of 1,217,564 inhabitants [26].

To determine the study population, we considered the total population served, defined as the population that had attended a consultation with any primary care centre professional at least once in the previous year (713,593 inhabitants) [26]. We considered the population with diabetes in the study areas and the population receiving home care [27].

### 2.3. Selection Criteria

Inclusion criteria: patients treated at a primary care centre for a chronic wound documented in their health record with one of the following diagnoses: PI, VLU, IU, DFU, lower-extremity ulcer of unknown aetiology (LEUUA) following ICD-10 classification [28] or other wounds (OW) present for more than a 6-week duration [29].

Exclusion criteria: people aged <15 years old at the time of data collection and people in institutions (nursing homes, care homes or hospitals).

### 2.4. Data Collection

All wounds treated in primary care centres between 16 April and 13 June 2013 were identified. Data from patients and wounds were collected from electronic health records. The wound diagnosis was retrieved directly from each patient’s medical history and was confirmed by direct patient assessment.

Collected data included the following sociodemographic and clinical variables of the patients: age (as a continuous categorical variable with the following age categories: >74 years, 65–74 years and <65 years); sex; aetiology of the chronic wound; predisposing drugs taken by the patient (glucocorticoids, chemotherapy and cardiotonics); Braden PI risk scale (categorical) [30]; wound stage (1–4 or unstageable) in the case of PI [31]; use of pressure relief surfaces (static surface, alternating pressure surface or no special surface) and use of compressive therapy. Wounds were characterised by frequency, site (lower leg, foot, heel, gluteus, scapula, sacrum, trochanter, occipital and other sites) and size (calculated by multiplying the length by width; if the surface area was greater than 10 cm^2^, a correction factor of 0.785 was applied) [32].

The total prevalence of chronic wounds was calculated using the following formula: people with a chronic wound in the study period, divided by the total number of people treated in primary care centres and multiplied by 100.

### 2.5. Statistical Methods

We conducted a univariate descriptive analysis to describe the population characteristics. Quantitative variables were described by the means and standard deviation (SD) and medians with interquartile range (IQR). For categorical variables, we calculated frequencies and percentages. The Kolmogorov–Smirnov test was used to determine the normality of the data distribution.

Associations between variables were estimated using the Chi-squared test in the case of categorical variables. To assess any differences in the distribution of quantitative variables among categories of a categorical variable, we used the nonparametric Kruskal–Wallis test with the post-hoc Mann–Whitney U test. All statistical analyses were performed using the SPSS (IBM Corp. Released 2015. IBM SPSS Statistics for Windows, Version 23.0. Armonk, NY, USA: IBM Corp).

Variables described as measured on patients were evaluated by the sample of patients attending wound treatment in primary care, whereas variables measured on wounds were described by the sample of wounds (without adjustment for intrapatient correlation).

## 3. Results

### 3.1. Patient Characteristics

We obtained 783 forms corresponding to patients with one or more active chronic wounds. The patients presented a total of 1061 wounds. The median of chronic wounds per patient was 1 (IQR: 1–1), and the median age in years was 79 (IQR: 70–86). The most prevalent age group was the one aged >74 years, at 64.9% (504). There was a higher proportion of women (57.5%) (Table 1).

### 3.2. Prevalence

Out of 713,593 individuals in the study population, 783 individuals were identified with chronic wounds, representing a prevalence of 0.11%; when adjusted for age, this prevalence was 0.56% in the >74 age group (508/90,334). By aetiology, VLU were the most prevalent, at 0.04% (286/713,593), followed by PI (0.03%, 236/713,593). In the >74 age group, the frequencies rose to 0.20% for VLU (182/90,334) and PI (180/90,334) alike (Table 2). By sex, women presented a higher prevalence of wounds 0.12% (450/389,568) (Table 3).

### 3.3. Aetiology, Age and Sex

We observed significant differences when chronic wound aetiology was related to age and sex (Table 4). The median age of patients with PI was 83 (IRQ 75–89.5). These results were higher than those obtained in patients with VLU (78, IRQ 69–86, *p* < 0.001), DFU (74, IRQ 61–82, *p* < 0.001), OW (72.5, IRQ 58–79, *p* < 0.001) and IU (76, IRQ 66–85, *p* = 0.029). The median age of patients with LEUUA was 80 (IRQ 72.5–86), which was also higher than that for patients with DFU (74, IQR: 61–82, *p* = 0.032) or those with OW (72.5, IRQ 58–79, *p* = 0.039).

The >74 age group presented the highest frequency of chronic wounds, accounting for 64.9% of cases. This group included the highest number of patients with PI (76.3%, *n* = 180), VLU (63.6%, *n* = 182) and LEUUA (70.5%, *n* = 62). In the 65–74 age group, the prevalence of the different aetiologies varied from 12.3% in patients with PI to 24.3% in patients with IU. Lastly, the <65 age group accounted for 31.1% of DFU and 33.3% of OW, significantly higher than for other aetiologies (*p* < 0.001 in the various post-hoc tests).

By sex, women presented a higher frequency of wounds (57.5%). This was observed for PI (56.4%, *n* = 133), VLU (62.6%, *n* = 179), LEUUA (76.1%, *n* = 67) and OW (60%, *n* = 18). Women were less prevalent in the case of IU (43.2%, *n* = 16) and DFU (34.9%, *n* = 37) (*p* < 0.001) (Table 4).

### 3.4. Aetiology and Size

Regarding the characteristics of chronic wounds, the median was 4 cm^2^ (IQR: 1–9.4). A comparison of aetiology showed that DFU, with a median of 1.0 cm^2^ (IQR: 0.4–4), were smaller than the other types of wound, which had a median size of 4–6 cm^2^ (*p* < 0.001). With a median of 4.0 cm^2^ (IQR: 1–9.4), PI were also significantly smaller than VLU, which presented a median size of 6.0 cm^2^ (IQR: 2–14.1) (*p* < 0.001).

### 3.5. Location of Chronic Wounds: Pressure Injury Stage and Risk

By anatomical site, 48% (*n* = 517) of all 1061 chronic wounds were in the supramalleolar region, 18% (*n* = 199) on the foot and 9.8% (*n* = 104) on the heel; thus, 77.3% (*n* = 820) of all chronic wounds occurred in these three locations. An analysis of PI location (Table 5) showed that the most frequent location was the sacrum (24.1%, *n* = 91), followed by the heel (23.8%, *n* = 90) and the foot (19.3%, *n* = 73), accounting for 67.2% (*n* = 254) of all PI. Stage 2 ulcers had the highest frequency (43.7%, *n* = 165).

Of the total number of patients with PI, 75.2% (*n* = 161) were classified as presenting some degree of risk, and of these, 59.8% (*n* = 128) presented a moderate or high risk. Twenty-two records were lost (Table 6).

## 4. Discussion

The aim of our study was to determine the prevalence of chronic wounds due to the lack of recent data on primary care settings in Spain, finding an observed prevalence of 0.11%. By aetiology, VLU presented the highest prevalence, of 0.04%, followed by PI, of 0.03%. Patients aged 75 or more were the most prevalent in wound treatment in primary care, accounting for 69.4% of cases. Among treated patients, this age group contained the highest frequency of PI (76.3%, *n* = 180), VLU (63.6%, *n* = 182) and LEUUA (70.5%, *n* = 62).

Only one study has analysed the prevalence of all chronic wounds in community settings in Spain [16], in contrast to the more frequent, up-to-date studies published on the prevalence of chronic wounds in hospitalised patients or nursing home residents.

Our results are comparable to those reported in other community studies applying a diverse range of methods. Our total prevalence of chronic wounds was 0.11%, similar to that found in Helsinki (0.10% in 2008 and 0.08% in 2016) [21] and in Ireland (0.10% in 2014) [10]. Three studies conducted in the UK have also reported similar results (0.09%–0.15%) [7,12,33].

The prevalence of PI in our study was 0.03% across the population and 0.23% in people aged over 64 years old. This result is similar to the findings reported in other community studies conducted across Europe [10,11,12,34].

In Spain, the most recent study on PI prevalence [13] reported a prevalence of 0.05% in the adult population, 0.27% in people aged over 65 years and 6.11% in patients treated at home. These figures are similar to those found in our study, with the exception of the results for people treated at home, since we observed a prevalence of 2.42%. With regard to the other PI characteristics, both the most frequent stage (stage 2, 43.7%) and the most frequent locations (sacrum, heel, foot, trochanter and gluteus) coincide with the results obtained in most published studies [11,13,20,34]. The percentage of patients in our study using pressure relief surfaces was lower (45%) than that reported in other studies (51%) [20].

In our study, the prevalence of VLU in the adult population, in people aged over 65 years and by sex was similar to the results obtained in other studies. For example, Hall and Srinivasaiah [12,20] estimated a prevalence of approximately 0.04% in the total population. Studies conducted closer to our study area have found similar distributions adjusted by sex and higher frequencies, ranging between 0.07% and 0.09% [16,17,19]. Other studies have reported a lower prevalence, from 0.01% to 0.03% [7,18]. The use of compression therapy in our study was low (19.9%) compared with the results obtained in previous studies (50%) [10,20]. Of particular note is the use of compression therapy in 14.8% of LU not diagnosed as venous.

In relation to LU, it was necessary to pool our results for VLU, IU, PI and LEUUA in order to compare them with those of other studies. The percentage of LU with respect to total wounds identified was 77%, higher than the 60% estimated in other studies [10,21]. The prevalence of LU was 0.06% (*n* = 411), similar to that reported by Ahmajärvi [21], but higher than the figures given in other studies, which have ranged from 0.04% to 0.05% [12,18,29,33,35], and lower than the 0.15% reported in the meta-analysis by Martinengo [8]. The very high figure found in this meta-analysis may have been due to the inclusion of a one-year cumulative prevalence study [36], which differed from our study design.

The prevalence of DFU was 0.01% in the total population and 0.16% in the population with diabetes, prompting caution with regard to reliability. This prevalence is similar to that reported in other, nonspecific studies on DFU, which have found prevalence ranging between 0.01% and 0.03% in the total population, and similar percentages of DFU with respect to the total of chronic wounds (13.5%) [10,12,16,21]. However, it is not possible to compare our results with those of more specific studies of DFU due to differences in the methods used and outcome variable studied. Some studies have reported a cumulative prevalence ranging between 0.08% and 2.9% in the population with diabetes [22,36,37,38,39]. These data confirm that patients with DFU present a different demographic and clinical profile to that of all other cases of chronic wounds, since these ulcers are more prevalent in men, occur at an earlier age of onset and have a smaller surface area [22]. Knowledge of the age of onset of the different chronic wounds would be useful to tailor preventive measures to age groups with a higher prevalence.

In our study, and in the majority of the literature, the frequency of ulcers is higher in women than men [12,23,24,33,36]. Our view is that such sex differential reflects the population pyramid in our country and across Europe. As women generally have a longer life expectancy, there are disproportionately represented in the over-65-years age stratum [40]. The higher the number of people over 65 years, the higher the number of chronic wounds. We adjusted the prevalence by sex, with a total prevalence of 0.10 in men and 0.12% in women.

When analysing the aetiology, a higher prevalence of LU was found in women (0.06%), which resonates with the published data [16,17,18,19]. Prevalence of DFU and IU was higher in men, which may be due to the relationship of this type of wound with cardiovascular risk, which is greater in men, and the fact they appear at younger ages [22,38,41].

Differences in results between studies are often attributed to methodological variability [10,12,13,21,42]. However, it is less common to cite this variability when the results are similar. Consequently, we suggest the need to develop standardised protocols for epidemiological studies of chronic wounds in the community in order to enable subsequent reliable comparisons.

One of the strengths of our study was that it was conducted within the context of a universal health care system with the participation of all Institut Català de la Salut primary care centres in the region, and it included the majority of patients receiving some type of health care. The percentage of people being treated increased with age, and this helped minimise selection bias in the age group with the highest prevalence of chronic wounds.

With regard to the implications for professional practice, our data could help inform the redesign of community health care models for patients with chronic wounds, facilitating a reduction in costs [2,3] and use of resources [11], and improve quality of life indicators [6].

Our results indicate the need to increase the use of alternating air pressure surfaces for PI and compression therapy for VLU. It would also be helpful to establish a network of nurses specialising in chronic wounds in primary care to support primary care teams and coordinate with hospital specialists in chronic wounds, in order to achieve integrated, effective and efficient health care.

### Limitations

This study also presents some limitations. For example, our sample did not include patients who did not attend their assigned public health care centres, because they received treatment either in nursing homes, in private institutions or exclusively in hospitals, and this may have led to an underestimation of the real prevalence in nonresidential community settings. With regard to the diagnostic process, it should also be borne in mind that this was based on the clinical opinion of the primary care team, and in many instances, the diagnosis was not subject to specialist assessment. Another possible limitation that might have affected the data obtained for DFU was a lack of awareness among professionals of the diagnostic process for these ulcers because the Diabetic Foot Hospital Unit did not exist at the time of the study and was only created later that same year.

## 5. Conclusions

The results obtained for chronic wound prevalence in 2013 in a community setting in the south of the province of Barcelona are consistent with those reported in previous similar studies conducted in Spain and elsewhere.

As with most studies that adjusted their variables for age and sex, we found that the prevalence of ulcers increased with age and was higher in women, except in the case of DFU and IU, which were more frequent in men.

The creation of an agreed methodology for epidemiological studies of chronic wounds in community settings would enable comparisons between future studies in different health care systems.

## Figures and Tables

**Table 1:** Patient characteristics.

Patient Characteristics	
Population assigned/treated, *n*/*n*	1,217,564/713,593
Patients with wounds, *n*	783
Age, years ^1^	
Mean (SD)	76.4 (14.0)
Median (IQR)	79 (70–86)
Age (years), *n* (%)	
<65	134 (17.1%)
65–74	141 (18%)
>74	508 (64.9%)
Sex, *n* (%)	
Male	333 (42.5%)
Female	450 (57.5%)
Aetiologies, *n* (%)	
Pressure Injury	236 (30.1%)
Venous ulcer	286 (36.5%)
Other leg ulcer	88 (11.2%)
Ischaemic ulcer	37 (4.7%)
Diabetic foot	106 (13.5%)
Other	30 (3.8%)
Special surface for pressure management, *n* (%)	
Static surface	57 (24.15%)
Alternating pressure surface	48 (20.34%)
No special surface	131 (55.51%)
Cardiotonic drugs, *n* (%)	
Yes	130 (16.6%)
Corticosteroid, *n* (%)	
Yes	54 (6.9%)
Chemotherapy, *n* (%)	
Yes	14 (1.8%)
Wounds, *n*	1061
Number of wounds per patient ^1^	
Mean (SD)	1.4 (0.8)
Median (IQR)	1 (1–1)
Surface area (cm^2^) ^1^	
Mean (SD)	10.5 (20.4)
Median (IQR)	4 (1–9.4)

^1^ This variable does not have a normal distribution.

**Table 2:** Prevalence of chronic wounds.

Aetiology	Age	Population	*n*	Prevalence	Confidence Interval
Pressure injury	<65	527,075	27	0.005%	(0.003–0.007)
	65–74	96,184	29	0.030%	(0.019–0.041)
	>74	90,334	180	0.199%	(0.17–0.228)
	^1^ HC	9734	236	2.424%	(2.118–2.73)
	Subtotal	713,593	236	0.033%	(0.029–0.037)
Venous ulcer	<65	527,075	46	0.009%	(0.006–0.012)
	65–74	96,184	58	0.060%	(0.045–0.075)
	>74	90,334	182	0.201%	(0.172–0.23)
	Subtotal	713,593	286	0.040%	(0.035–0.045)
Other leg ulcer	<65	527,075	10	0.002%	(0.001–0.003)
	65–74	96,184	16	0.017%	(0.009–0.025)
	>74	90,334	62	0.069%	(0.052–0.086)
	Subtotal	713,593	88	0.012%	(0.009–0.015)
Ischaemic ulcer	<65	527,075	8	0.002%	(0.001–0.003)
	65–74	96,184	9	0.009%	(0.003–0.015)
	>74	90,334	20	0.022%	(0.012–0.032)
	Subtotal	713,593	37	0.005%	(0.003–0.007)
Diabetic foot	<65	527,075	33	0.006%	(0.004–0.008)
	65–74	96,184	23	0.024%	(0.014–0.034)
	>74	90,334	50	0.055%	(0.040–0.070)
	Subtotal	713,593	106	0.015%	(0.012–0.018)
	^2^ DM	67,815	106	0.156%	(0.126–0.186)
Other	<65	527,075	10	0.002%	(0.001–0.003)
	65–74	96,184	6	0.006%	(0.001–0.011)
	>74	90,334	14	0.015%	(0.007–0.023)
	Subtotal	713,593	30	0.004%	(0.003–0.005)
Subtotal	<65	527,075	134	0.025%	(0.021–0.029)
	65–74	96,184	141	0.147%	(0.123–0.171)
	>74	90,334	508	0.562%	(0.513–0.611)
	Subtotal	713,593	783	0.110%	(0.102–0.118)

^1^ HC: patients receiving home care. ^2^ DM: population with diabetes mellitus.

**Table 3:** Prevalence of chronic wounds. Adjusted by sex.

Aetiology	Sex	Population	*n*	Prevalence	Confidence Interval
Pressure injury	Female	389,568	133	0.034%	(0.028%–0.040%)
	Male	324,025	103	0.032%	(0.026%–0.038%)
Venous ulcer	Female	389,568	179	0.046%	(0.039%–0.053%)
	Male	324,025	107	0.033%	(0.027%–0.039%)
Other leg ulcer	Female	389,568	67	0.017%	(0.013%–0.021%)
	Male	324,025	21	0.006%	(0.004%–0.009%)
Total leg ulcer (nonischaemic)	Female	389,568	246	0.063%	(0.055%–0.071%)
	Male	324,025	128	0.040%	(0.033%–0.046%)
Ischaemic ulcer	Female	389,568	16	0.004%	(0.002%–0.006%)
	Male	324,025	21	0.006%	(0.004%–0.009%)
Diabetic foot	Female	389,568	37	0.009%	(0.006%–0.013%)
	Male	324,025	69	0.021%	(0.016%–0.026%)
	Female	389,568	18	0.005%	(0.002%–0.007%)
Other	Male	324,025	12	0.004%	(0.002%–0.006%)
Subtotal	Female	389,568	450	0.116%	(0.105%–0.126%)
	Male	324,025	333	0.103%	(0.092%–0.114%)

**Table 4:** Wound characteristics.

	Pressure Injury	Venous Ulcer	Other Leg Ulcer	Ischaemic Ulcer	Diabetic Foot Ulcer	Other Wounds	Total	Sig
Patients, *n*	236	286	88	37	106	30	783	
Age (years) ^3^								<0.001 ^1^
Mean (SD)	80 (14.7)	75.8 (13.6)	77.5 (12.7)	74.8 (11.8)	71.8 (13.5)	69.2 (13.7)	76.4 (14.0)	
Median (IQR)	83 (75–89.5)	78 (69–86)	80 (72.5–86)	76 (66–85)	74 (61–82)	72.5 (58–79)	79 (70–86)	
Age (years), *n* (%)								<0.001 ^2^
<65	27 (11.4%)	46 (16.1%)	10 (11.4%)	8 (21.6%)	33 (31.1%)	10 (33.3%)	134 (17.1%)	
65–74	29 (12.3%)	58 (20.3%)	16 (18.2%)	9 (24.3%)	23 (21.7%)	6 (20.0%)	141 (18%)	
>74	180 (76.3%)	182 (63.6%)	62 (70.5%)	20 (54.1%)	50 (47.2%)	14 (46.7%)	508 (64.9%)	
Sex, *n* (%)								<0.001 ^2^
Male	103 (43.6%)	107 (37.4%)	21 (23.9%)	21 (56.8%)	69 (65.1%)	12 (40.0%)	333 (42.5%)	
Female	133 (56.4%)	179 (62.6%)	67 (76.1%)	16 (43.2%)	37 (34.9%)	18 (60.0%)	450 (57.5%)	
Cardiotonic drugs, *n* (%)								0.181 ^2^
Yes	29 (12.3%%)	52 (18.2%)	15 (17.0%)	7 (18.9%)	24 (22.6%)	3 (10.0%)	130 (16.6%)	
No	207 (87.7%)	234 (81.8%)	73 (83.0%)	30 (81.1%)	82 (77.4%)	27 (90.0%)	653 (83.4%)	
Corticosteroid, *n* (%)								0.217 ^2^
Yes	9 (3.8%)	25 (8.7%)	7 (8.0%)	1 (2.7%)	9 (8.5%)	3 (10.0%)	54 (6.9%)	
No	227 (96.2%)	261 (91.3%)	81 (92.0%)	36 (97.3%)	97 (91.5%)	27 (90.0%)	729 (93.1%)	
Chemotherapy, *n* (%)								
Yes	5 (2.1%)	3 (1.0%)	1 (1.1%)	1 (2.7%)	0 (0.0%)	4 (13.3%)	14 (1.8%)	<0.001 ^2^
No	231 (97.9%)	283 (99.0%)	87 (98.9%)	36 (97.3%)	106 (100.0%)	26 (86.7%)	769 (98.2%)	
Compression therapy	-	57 (19.9%)	13 (14.8%)	-	-	-		
Wounds *n*	*n* = 378	*n* = 364	*n* = 107	*n* = 51	*n* = 124	*n* = 37	*n* = 1061	
Surface area (cm^2^) ^3^								<0.001 ^1^
Mean (SD)	7.8 (12.6)	14.2 (26)	11.1 (19.9)	16.2 (30.5)	4.3 (7.1)	13.9 (25.9)	10.5 (20.4)	
Median (IQR)	4 (1–9.4)	6 (2–14.1)	6 (1–11.8)	4 (1–14.1)	1 (0.4–4)	5 (1–12.6)	4 (1–9.4)	

^1^ Kruskal–Wallis. ^2^ Chi-squared. ^3^ This variable does not have a normal distribution.

**Table 5:** Pressure injury stage and location.

	Stage 1	Stage 2	Stage 3	Stage 4	Unstageable	Total
Lower leg	2	6	1	0	4	13 (3.4%)
Foot	18	35	17	0	3	73 (19.3%)
Heel	15	50	20	3	2	90 (23.8%)
Gluteus	12	9	3	2	0	26 (6.9%)
Scapula	0	1	1	1	0	3 (0.8%)
Sacrum	15	35	25	14	2	91 (24.1%)
Trochanter	11	23	11	9	6	60 (15.9%)
Other	8	6	6	1	0	21 (5.6%)
Occipital	1	0	0	0	0	1 (0.3%)
Total	82 (21.7%)	165 (43.7%)	84 (22.2%)	30 (7.9%)	17 (4.5%)	378 (100%)

**Table 6:** Pressure injury Braden risk.

		Total	
		*n*	%
Braden risk	No risk (>18)	53	24.8%
	At risk (15–18)	33	15.4%
	Moderate risk (13–14)	48	22.4%
	High risk (10–12)	53	24.8%
	Very high risk (<10)	27	12.6%
	Total	214	100.0%

## Data Availability

Data available on request due to ethical restrictions.
